# Buckling-induced sound production in the aeroelastic tymbals of *Yponomeuta*

**DOI:** 10.1073/pnas.2313549121

**Published:** 2024-02-05

**Authors:** Hernaldo Mendoza Nava, Marc W. Holderied, Alberto Pirrera, Rainer M. J. Groh

**Affiliations:** ^a^Bristol Composites Institute, School of Civil, Aerospace & Design Engineering, University of Bristol, Bristol BS8 1TR, United Kingdom; ^b^School of Biological Sciences, University of Bristol, Bristol BS8 1TQ, United Kingdom

**Keywords:** biomechanics, bioinspiration, bioacoustics, snap-through, stability

## Abstract

The loss of elastic stability (buckling) can lead to catastrophic failure in the context of traditional engineering structures. Conversely, in nature, buckling often serves a desirable function, such as in the prey-trapping mechanism of the Venus fly trap (*Dionaea muscipula*). This paper investigates the buckling-enabled sound production in the wingbeat-powered (aeroelastic) tymbals of *Yponomeuta* moths. The hindwings of *Yponomeuta* possess a striated band of ridges that snap through sequentially during the up- and downstroke of the wingbeat cycle—a process reminiscent of cellular buckling in compressed slender shells. As a result, bursts of ultrasonic clicks are produced that deter predators (i.e. bats). Using various biological and mechanical characterization techniques, we show that wing camber changes during the wingbeat cycle act as the single actuation mechanism that causes buckling to propagate sequentially through each stria on the tymbal. The snap-through of each stria excites a bald patch of the wing’s membrane, thereby amplifying sound pressure levels and radiating sound at the resonant frequencies of the patch. In addition, the interaction of phased tymbal clicks from the two wings enhances the directivity of the acoustic signal strength, suggesting an improvement in acoustic protection. These findings unveil the acousto-mechanics of *Yponomeuta* tymbals and uncover their buckling-driven evolutionary origin. We anticipate that through bioinspiration, aeroelastic tymbals will encourage novel developments in the context of multi-stable morphing structures, acoustic structural monitoring, and soft robotics.

## Significance

Moths of the genus *Yponomeuta* possess wingbeat-powered tymbals that use sequential snap-through instabilities for sound production. The resulting bursts of clicks serve as an ultrasound protection mechanism against bats. Using detailed biological and mechanical characterization, we map the intricate morphology of aeroelastic tymbals and use simple models from structural engineering to describe the mechanics and acoustics of sequential, buckling-driven sound production. In the past, elastic instabilities observed in the natural world, such as in the Venus fly trap, have motivated engineers to develop novel bioinspired soft robots and morphing structures. In this vein, exploiting sequential buckling could lead to novel shape-changing structures, where buckling-induced sound production offers additional and currently unexplored functionality.

Moths have evolved and developed varying acoustic defense mechanisms to counteract echolocating bats that prey upon them. Recent research has shown that certain moth species benefit from active or passive defense strategies to deter bats (1) including decoy echoes from wingtip ripples (2), acoustic stealth camouflage (3–5), and the emission of acoustic signals. Antibat sounds produced by moths may serve as warning signals (i.e., acoustic aposematism) (6, 7) or jam a bat’s echolocation systems (8, 9). Extensive research has shown, for example, that tiger moths (*Arctiine*) produce bat-deterring ultrasounds by buckling or snapping their so-called tymbal organs (8, 10, 11)—a cuticular membrane (often striated on one side) located at the moth’s thorax. It is now well established that the thoracic tymbals of tiger moths are buckled deliberately by muscular action, usually in response to bat echolocation calls (12). For the characteristic rippling sound of striated tymbal organs to be produced, it is generally assumed that snap-through buckling of the striae occurs progressively. For instance, Corcoran et al. (8) have shown that in tiger moths buckling propagates through individual striae, leading to a first burst of clicks, followed by a second burst when the buckling deformations are reversed.

In other moth species, tymbal organs are found in the abdomen, tegula, or the wings (13). For example, it has been reported that ermine moths (*Yponomeuta*) possess wingbeat-powered tymbals that buckle continuously during flight. As a result, *Yponomeuta* tymbals have been termed “aeroelastic tymbals” (11). Recent publications show that wing tymbals are present in several species of microlepidoptera (14). Interestingly though, research on tymbal biomechanics is scarce and the mechanical factors driving click production are yet to be determined. This paper focuses on explaining how tymbal clicks are actuated (by claval rotation), produced (by snap-through of individual facets of a striated band), facilitated (by graded material properties), and amplified (by a bald patch adjacent to the striated band).

Wing tymbals equip the deaf *Yponomeuta* species with a passive and perpetual acoustic defense mechanism (11). In particular, two bursts of ultrasonic clicks are emitted for every wingbeat cycle. One burst takes place during the upstroke stage (the upper burst), when the wing pronates, while the second burst occurs during the downstroke (the lower burst), when the wing supinates. Each burst coincides temporally with the occurrence of hindwing flexion along the claval flexion line (11), although a causal mechanism tying hindwing flexion to click production is yet to be determined. Claval flexion is driven by an interaction of aerodynamic, elastic, and inertial forces and enabled by the presence of compliant strips of cuticle acting as flexion and folding lines (15). One objective of the present paper is to provide experimental evidence that claval flexion is indeed the mechanical stimulus that triggers the burst of clicks.

It is also generally accepted that backing air sacs in thoracic tymbals are used as sound amplification devices (16). However, wing-mounted aeroelastic tymbals do not possess such an advantageous sound amplification feature. Nonetheless, the radiation distance of ultrasonic signals emitted by *Yponomeuta* projects well into the far field and is equal to the sensing distance that a bat can perceive through echolocation (11). Hence, alongside the line of enquiry into the biomechanical actuation of *Yponomeuta* wing tymbals, there are also questions on the mechanisms responsible for the strength and directivity of their acoustic signal. Answering these questions is another objective of the present study.

By direct manipulation of the aeroelastic tymbal, the present research explores how the striations can produce clicks. The buckling dynamics of the aeroelastic tymbal is also explored by conducting detailed finite element (FE) simulations at a single-stria level, aiming to simplify and explain the sequential buckling behavior that leads to a burst of clicks. In addition, we investigate how aeroelastic tymbals use structural resonance for sound production and report the conditions for which the acoustic field is directionally enhanced for optimal protection.

This contribution provides insights into buckling-driven sound production of wing tymbals. We show that aeroelastic tymbals are intrinsically complex structures whose response is governed by the interplay of intricate geometric form and graded material properties, likely a result of morphogenetic development (17). Moreover, our research encourages pioneering developments in the field of morphing structures and micro-electromechanical systems (MEMS), where sequential buckling, inspired by aeroelastic tymbals, could expand current systems based on bistability toward more complex multi-stable responses. In this manner, *Yponomeuta* stand out as a novel source of inspiration for the design of shape-changing structures.

## Aeroelastic Tymbals Are Complex Graded Cuticular Structures

**Fig. 1.**
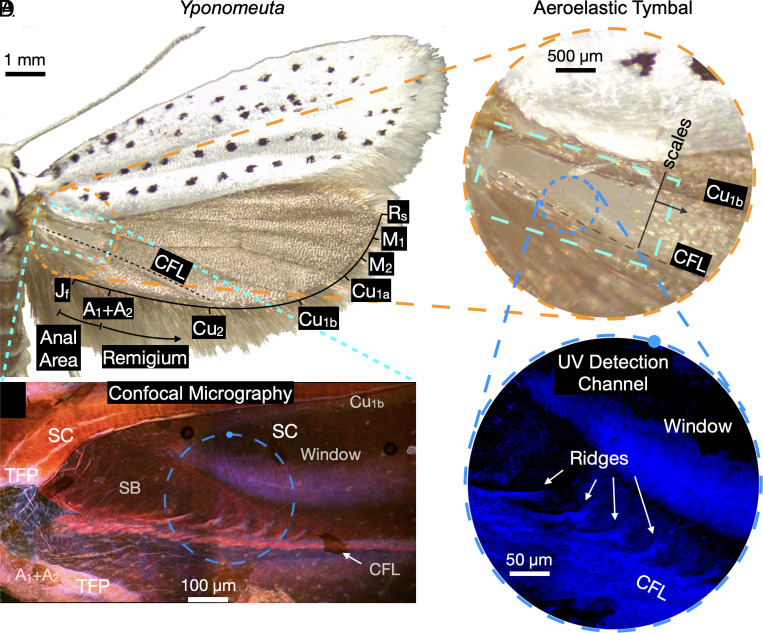
The aeroelastic tymbal of *Yponomeuta*. (*A*) Stereo micrograph of a pinned *Yponomeuta* (dorsal view), the dashed orange circle indicates the magnified area displayed in *B* and the light-blue dashed rectangle indicates the area of the confocal micrograph in *C*. (*B*) Aeroelastic tymbal, the dashed blue circle indicates the region of the tymbal shown in the confocal micrographs (*C* and *D*) the solid dot on the dashed line indicates rotation of the confocal micrograph. (*C*) Confocal microscopy of the aeroelastic tymbal (maximum intensity projection of the four detection channels). (*D*) Maximum intensity projection of the UV channel. CFL—claval lexion line, SB—striated band, SC—sclerotized cuticle, TFP—tough flexible part.

Fig. 1*A* indicates the hindwing venation of *Yponomeuta*. The aeroelastic tymbal (Fig. 1*B*) is located between the Cu_1*b*_ vein and the claval flexion line, which lies adjacent to the Cu_2_ vein. Insect wings are essentially constituted of cuticle, a natural composite material which consists of orthotropic layers of chitin micro-fibrils embedded in a protein matrix (18). Flexion lines are stripes of flexible cuticle that can act as hinges or joints and are crucial for camber shape adaptation (19). Resilin, a rubber-like material that can be found as an additional constituent in cuticle materials, is typically detected along wing folding and flexion lines, for instance, on the wings of honeybees and earwigs (20, 21), and it has been reported to be present in the abdominal tymbals of Noctuoidea (22). Resilin is characterized by its low energy loss under dynamic loading (23) and can be observed in cuticle materials using laser scanning confocal microscopy (LSCM) (24, 25).

Fig. 1*C* shows a confocal micrograph of the aeroelastic tymbal, which exhibits the combined autofluorescence at different excitation wavelengths (excitation wavelength: 405 (blue), 488 (green), 561 (red) and 633 nm (red), emission: 420 to 480, 495 to 551, 568 to 700 and 650 to 790 nm, respectively). While confocal microscopy has been used to characterize insect cuticle (18, 24) and here uncovers the graded material distribution of aeroelastic tymbals. Fig. 1*D* zooms in on a smaller region of the tymbal, exposed exclusively to UV light (405 nm wavelength). The blue coloration corresponds to the characteristic autofluorescence of resilin and indicates the claval flexion line, located inside the scaleless window toward the striated band, as well as the ridges of the striated band. The combined autofluorescence image reveals the level of sclerotization (24), where, for example, the red-dominated coloration at the Cu_1*b*_ vein (which is a main load-bearing structure) indicates a high sclerotization. Conversely, the wing membrane shows multiple levels of sclerotization. The typical coloration hues of tough cuticle, denoted by a pink coloration, occur at the positions of veins A_1_ and A_2_. The striated band shows hues varying from purple to pink at the band’s base and strong pink along the ridges. Interestingly, a purple to pink coloration is displayed on the scaleless window toward the striated band, which indicates a more compliant region. In contrast, the rest of the window (at the interface between the striated band and the window and on the segment of the window toward the Cu_1*b*_ vein) is shown as a darker area, which implies a thinner thickness with a higher sclerotization. The detailed visualization of the aeroelastic tymbal using LSCM exhibits the complex graded cuticular properties of the wing’s membrane.

The biological characterization of the striated band and the adjacent wing provides ample information to define approaches to translate the morphological complexity into simplified engineering analogues to elucidate the structure’s local dynamics.

## Displacement Controlled Snap-Down Instabilities Are Triggered by Claval Rotation

While it has been reported that *Yponomeuta* produce two bursts of clicks throughout each stroke cycle (11), this phenomenological observation has not been accompanied by an explanation of the mechanism triggering the bursts. In addressing this gap, our initial hypothesis considered clap-and-fling Weis-Fogh mechanism (26)—observed in supplementary video M1 of ref. 11—as a potential mechanism. However, high-speed videos of different specimens of *Y. malinellus* and *Y. cagnagella* revealed that the burst is produced regardless of the clapping (*SI Appendix*, Movie S1, 27). As an alternative explanation, we propose that the upper burst occurs due to the progressive unfolding of the hindwings throughout the upstroke stage. The dynamic camber change enabled by the compliant claval flexion line (15) acts as a trigger to initiate local buckling of the tymbal stria. Our observations show that a dorsal folding movement of the anal region of the hindwing, against the remigium along the claval flexion line, causes the lower burst. Our observations also suggest that the unfolding of the hindwing between the remigium and the anal regions during the upstroke produces the upper burst. We refer to this folding movement as claval rotation and its occurrence during the up- and downstroke wingbeat cycle explains both bursts of clicks.

Upon physical manipulation of a hindwing, or, more specifically, upon application of a negative claval rotation (folding or flexing movement), we observe that it is possible to buckle the striations and induce a burst of clicks that can be clearly heard through a bat detector. Through a stereo microscope, we observe that the striations snap in sequence—a behavior reminiscent of cellular buckling (28) in slender shells or architected materials where one snap-through instability triggers a sequence of further instabilities. Similarly, sequential snap-through of the striated band progresses in an ordered fashion when subjected to claval rotation. A negative claval rotation creates a snapping sequence that starts from the distal end of the striated band and progresses toward the base of the hindwing. This sequence propagates in the opposite direction during positive claval rotation (*SI Appendix*, Movie S2, 27). We observe that the location at which the striations snap-through corresponds to the region of reduced thickness at the interface between the striated band and the window (Fig. 1*C*) where we have also identified a compliant resilin band (Fig. 1*D*).

## Spatially Graded Bending Stiffness Is Key to Enable a Buckling Sequence

**Fig. 2.**
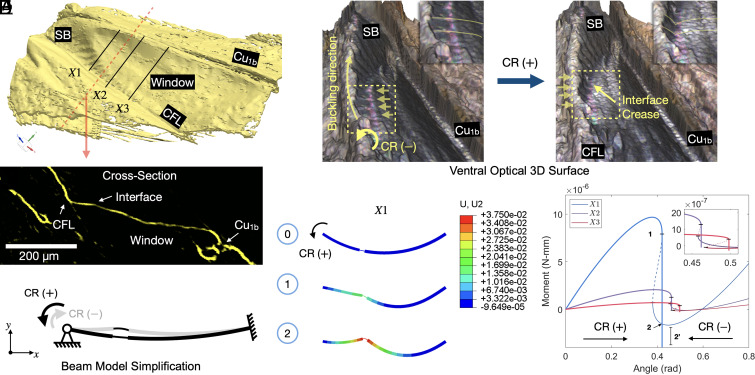
Morphology of the aeroelastic tymbal and buckling mechanism. (*A*) micro-CT scan of a right-hindwing aeroelastic tymbal (tilted dorsal view), with the cross-section indicated by the red dashed line shown in *C*. (*B*) Optical 3D surface of the ventral side of the tymbal when the hindwing is at a relaxed position (*Left* frame) and when the hindwing is extended (*Right* frame). The dashed frames indicate the enlarged regions in the top right corner of each picture, highlighting the morphological changes between the buckled and unbuckled configurations. (*C*) Cross-section of the aeroelastic tymbal indicated by the red dashed line in *A*. (*D*) Simplified model of a cross-section of the aeroelastic tymbal based on a curved beam with a compliant segment. An angular displacement is applied at the pinned left end of the beam and a fully fixed (clamped) boundary condition is enforced at the right end. (*E*) Snap-down sequence of the beam from station X1 in A (*Right*), and equilibrium paths of the three beams under angular displacement (*Right*). The dashed lines in *E* indicate unstable equilibria, and the black horizontal markers indicate the limit points for snap-down and snap-upward instabilities. CR—claval rotation, U2—vertical displacement in mm (Y-axis).

Detailed imaging of the aeroelastic tymbal using micro-computed tomography (micro-CT, Fig. 2*A*) reveals that the aeroelastic tymbal is doubly curved. Hence, aeroelastic tymbals consist of a smooth doubly curved membrane (window) and a striated band (or band of microtymbals) running alongside the claval flexion line. Dorsally, the striated band can be described as a series of sharp ridges and broader valleys. The curvature of the window diminishes at a certain distance ahead of the area where the scales are visible (Fig. 1*B*), which we propose as the spanwise limit of the aeroelastic tymbal.

In Fig. 2*B*, we show an optical 3D surface measurement of a hindwing at the aeroelastic tymbal. The left picture shows the hindwing in a resting position (no manipulation), where the horizontal arrows indicate buckled striations. In the right picture, the hindwing is subjected to positive claval rotation by constraining the remigium and creating a lever movement by pushing the anal area of the wing upward using an insect pin mounted on a micromanipulator (*SI Appendix*, Fig. S1). As a result, a crease can be observed at the interface (completely bent interface) between the striated band and the window. These images support our previous acoustic findings from the bat detector that it is the claval rotation that causes bending to occur at the interface of the striated band and the window and that this local folding leads to buckling instabilities in the stria.

We subsequently measure the transverse (chordwise) curvature of an aeroelastic tymbal from the micro-CT scan at the three locations X1–X3 indicated in Fig. 2*A*. The measured cross-sections are used to build simple individual beam models (Fig. 2 *C* and *D*) to illustrate the buckling behavior of the aeroelastic tymbal. Despite their simplicity, these representative structural models capture the key physics of the phenomenon, i.e., snap-through buckling due to a boundary rotation. We set the initial configuration as the condition when the hindwing is flexed along the claval flexion line and the anal region is folded toward the remigium (continuous beam curvature). The buckled configuration then corresponds to the extended hindwing state (bent interface). The curved beam models are subjected to positive claval rotation (Fig. 2*E*) as the trigger mechanism to induce snap-through buckling. The equilibrium paths and the dynamic solutions are plotted in Fig. 2*E*, *Right*, where it is shown that, for the three cases considered, when subjected to controlled angular rotation, a snap-down instability is produced (1 to 2 in Fig. 2*E*). The relative location of the three limit points (and the resulting dynamic snap-down) on each of the three equilibrium paths indicates sequential snapping, with each snap occurring at an incremental value of angular rotation. In our proposed models, this behavior stems from tuning the bending stiffness of the arch sections by applying thickness reductions (Table 1). Finally, we compute the average nodal displacement ($\overset{¯}{U}$) of the three beams during snap-down along the y-axis where beam X3 shows the minimum averaged displacement ${\overset{¯}{U}}_{\text{min}}=3\;\mu$m and beam X1 the maximum displacement ${\overset{¯}{U}}_{\text{max}}=5\;\mu$m. These snap-down values are later used to estimate the sound pressure levels that are expected from displacing a column of fluid by ${\overset{¯}{U}}_{\text{min}}$ and ${\overset{¯}{U}}_{\text{max}}$. We have also compared the peak snap-through displacement of each of the three models (at Stations X1–X3) with the maximum morphological changes obtained by mechanically buckling and relaxing the aeroelastic tymbal under an Alicona InfiniteFocus G5 microscope (Alicona Imaging GmbH, X-Y step size: 2.6 μm and Z step size: 5 μm; see Fig. 2*B*). The modeled response and experimental measurements are within the same order of magnitude (≈10μm; see *SI Appendix*, Figs. S2 and S3 and Table S1). By neglecting the stiffening influence of curvature in the second dimension (perpendicular to the beam axes), the 1D models are generally more compliant than the actual tymbal structure.

**Table 1.** Dimensions and parameters of the arch models (see *SI Appendix*, Fig. S6 for model schematic)

Variable name	Station X1	Station X2	Station X3	Units
Total base	237	288	329	μm
Chord	169	234	284	μm
Radius	209	288	348	μm
*t*_SB_	6	6	7	μm
*t*_hinge_	1.02	1.05	0.5	μm
*t*_win_	5	3	2	μm
Length hinge	10	10	10	μm

In summary, our beam models, the corresponding equilibrium paths, and the positions of the limit points thereon, show that, when the hindwing is subjected to positive claval rotation (extension of the hindwing), the striations buckle starting from the most proximal station to the body (Station X1) toward the most distal station (Station X3). The sequence of buckles is reversed when a negative claval rotation is applied. This interpretation of the snap-down sequence also agrees with the experimentally observed buckling sequence in (*SI Appendix*, Movie S2, 27).

## Buckling Excites the Resonant Frequencies of Aeroelastic Tymbals

**Fig. 3.**
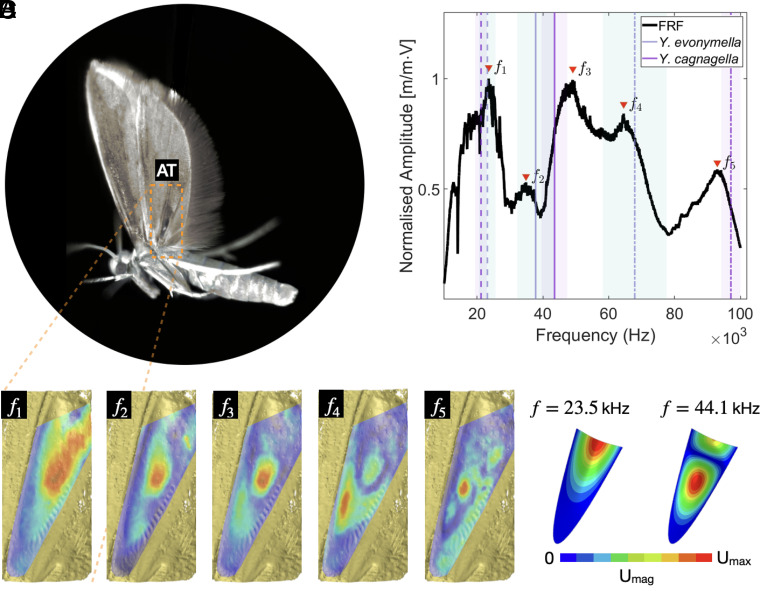
Dynamic response of the aeroelastic tymbal. (*A*) Lateral view of *Yponomeuta* during upstroke. (*B*) FRF of the averaged amplitude (black line) over the area delimited by the aeroelastic tymbal; the peak amplitudes are indicated by the inverted red triangles. The acoustic frequencies are indicated in *B* as mean acoustic peak (continuous line), high (dash-dotted line) and low frequencies (dashed line), and the translucent areas denote the SD of *Y. evonymella* (green) and *Y. cagnagella* (purple) (11). (*C*) Modal shapes obtained through scanning LDV. (*D*) Modal shapes obtained through FE. U—displacement in the Y-axis.

It has been speculated that the clear patches on the hindwings of ermine moths could act as resonators (29). Crickets, for example, radiate sound from the vibration of wing membrane cells (i.e. the harp region), that are excited due to the stridulation between the wings, when the plectrum (i.e., a scraper) strikes back and forth a serrated structure (file), both located on opposite edges of the wings (30). This phenomenon gives rise to the well-known pure-tone cricket songs. Here, we demonstrate that the sound radiated by the aeroelastic tymbal does indeed correspond to the resonant frequencies of the tymbal membrane, specifically of the clear window patch of the hindwing. We subject an ablated hindwing in the upstroke position (Fig. 3*A*) to mechanical vibrations by mounting it on a piezo-ceramic column (*SI Appendix*, Fig. S4) and measure the dynamic response using scanning laser Doppler vibrometry (LDV). The averaged mechanical frequency response function (FRF) is shown in Fig. 3*B*, as well as the acoustic frequencies of tethered moths measured experimentally by O’Reilly et al. (11). The frequency peaks are indicated with red markers in Fig. 3*B* at 23.5, 34.9, 49.1, 64.5, and 93.0 kHz, denoting the natural frequencies $f_{1}$ to $f_{5}$, respectively. Comparing the LDV measurements to the acoustic peak, low and high frequencies (highest and lowest frequency 15 dB below the amplitude of the peak frequency) of the same species in tethered flight (*Y. cagnagella*, $f_{\;\text{low}}=21.2\pm 1.8$, $f_{\;\text{peak}}=43.5\pm 3.9$, and $f_{\;\text{high}}=97.1\pm 2.9$ kHz), the first ($f_{1}$), third ($f_{3}$), and fifth ($f_{5}$) natural frequencies closely match the acoustic frequencies reported by O’Reilly et al. (11). The small differences can readily be explained by the fact that the wing was tested in a static position, while the actual tymbal during flight undergoes changes in strain after each stria snaps, which will affect the fundamental frequencies of the connected window. The modal vibration shapes (modes 1 to 5) are shown in Fig. 3*C* and reveal that the highest amplitude deformations at the resonant frequencies are restricted to the scale-free area of the tymbal. From these results, we suggest that the wing acts as a baffle boundary, where individual snaps act as impulse loads that excite the resonant frequencies of the window membrane.

## Coincident Clicks Increase the Acoustic Protection Zone in Yponomeuta

The clear patches bounding aeroelastic tymbals resonate to amplify the strength of clicking sounds. The experimental results reported by O’Reilly et al. (11) demonstrate that the acoustic field of the emitted clicks is near omnidirectional. In fact, if we model the tymbal area ($A_{\;\text{tymbal}}=2.55\times 10^{-7}\;$m^2^) as a baffled circular source, then, for the previously measured acoustic frequencies of interest ($f_{\;\text{low}}$, $f_{\;\text{peak}}$, and $f_{\;\text{high}}$), the ka parameter (ratio of the size of a source with respect to the wavelength λ; k— wavenumber, k=1/λ, a—radius of the source) is $k_{\;\text{low}}a\approx 0.11$, $k_{\;\text{peak}}a\approx 0.23$ and $k_{\;\text{high}}a\approx 0.51$. An equivalent ka parameter value of ka<1 corresponds to an omnidirectional sound radiation pattern (31), thereby satisfying the condition for omnidirectionality based on the assumption that the tymbal behaves as a baffled circular source.

However, the dorsal and ventral surfaces of the tymbal radiate sound in opposite phase, while being separated only by the finite acoustic boundaries created by the wings. Hence, each aeroelastic tymbal radiates sound as a dipole (no pressure in the parallel plane to the source (32)) instead, causing the sound to be radiated as single lobes normal to the wing surfaces during the upper and lower bursts. This would hinder the production of a strong acoustic field in the plane parallel to the wings, thereby indicating that other or additional mechanisms may be involved; for instance, acoustic reflections, simultaneous clicks, or favorable modal shapes that lead to the emission of acoustic waves parallel to the wings’ plane.

**Fig. 4.**
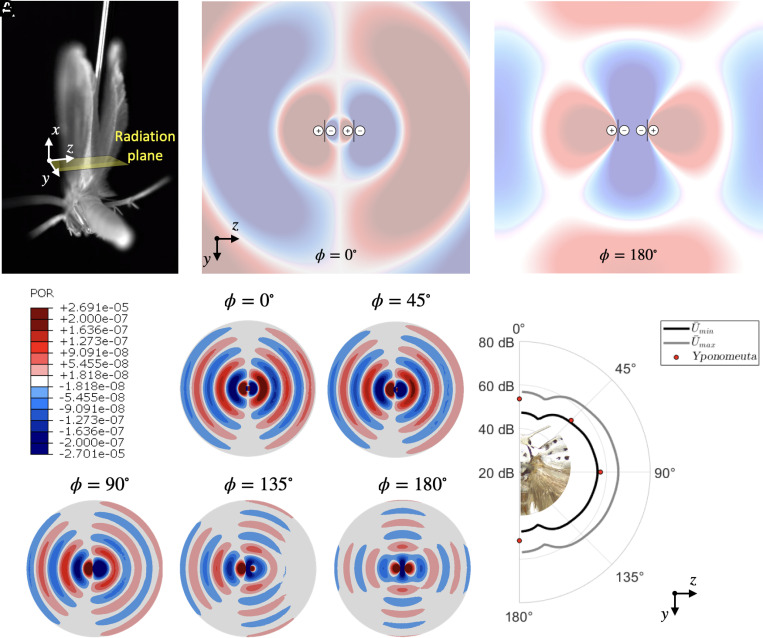
Sound radiation during the upstroke stage. (*A*) Photograph of a tethered *Yponomeuta* during upstroke. (*B*) Linear quadrupole comprising two dipoles radiating sound pressure at equal ($\phi =0^{{}^{\circ}}$) and opposite phase ($\phi =180^{{}^{\circ}}$). (*C*) Acoustic field of the plate model when the right plate has a phase of 0, 45, 90, 135, and $180^{{}^{\circ}}$. (*D*) Source level in the far field (at a reference distance r=0.1m, measured from the dorsal position), when the plates vibrate at ${\overset{¯}{U}}_{\text{min}}$ and ${\overset{¯}{U}}_{\text{max}}$ taken from the beam models. The red dots in D are reported SL values at r=0.1m (11).

Here, we address this issue by investigating the sound radiation of the wings in the upstroke position (Fig. 4*A*) using two different models. First, we assume that the tymbals radiate sound as point sources. Second, we implement a finite-element model of two vibrating plates on rigid boundaries that emulate the two wings.

Fig. 4*B* shows the acoustic field radiated by four point sources, which correspond to the top and bottom surfaces of the tymbal on each wing. Each pair of sources radiates at opposite phase, acting as dipoles, separated by the measured distance between the hindwings’ base joints (d=1.4mm, otherwise the width of the thorax at the position of the wing joints) when the wings are in a parallel vertical position. In the absence of any rigid boundary or baffle, the two dipoles form a linear quadrupole (33). When the two dipoles radiate sound at the same-phase condition ($\phi =0^{{}^{\circ}}$), a dipole-radiating field is obtained due to the acoustic cancelation between the mid sources. Conversely, when the two dipoles radiate at the opposite phase ($\phi =180^{{}^{\circ}}$), a frontal field is produced as a result of a stronger field combined from the mid sources.

In the case of the double-plate model (*SI Appendix*, Fig. S5), first, the Young’s modulus is adjusted to match the fundamental frequency of the tymbal (E=1.81GPa). Then, a modal analysis is carried out, where the first and second resonant frequencies and modal shapes agree with those obtained numerically (Fig. 3*D*). We subsequently assume that the plates vibrate at f=44.1kHz at the minimum and maximum averaged displacements obtained from the beam models discussed previously. Fig. 4*C* shows the pressure field of the vibrating plates from the equivalent upper view (frontal plane) of the moth during the upstroke, when the two sources radiate sound simultaneously with the same phase ($\phi =0^{{}^{\circ}}$) and when the right source vibrates at $\phi =\{45^{{}^{\circ}},90^{{}^{\circ}},135^{{}^{\circ}},180^{{}^{\circ}}\}$. The acoustic field at $\phi =0^{{}^{\circ}}$ resembles a dipole source, while at a phase condition of $\phi =180^{{}^{\circ}}$ the frontal field is produced in conformity with the linear quadrupole.

Finally, we compute the source level (SL) at the reference distance r=0.1m as reported in previous experiments (11). Fig. 4*D* shows the limit threshold of the SL from all phase conditions at minimum and maximum vibrating amplitude, where the red circular markers indicate the source levels reported by O’Reilly et al. (11). These measurements fall within the range predicted by our coupled structural-acoustic finite element models, showing excellent correlations between models and experiments given the complex geometry and multi-physics of the phenomenon.

## Discussion

Aeroelastic tymbals are complex, architected biological structures, where cuticular graded properties are essential for buckling-driven sound production and the ability to withstand the ensuing instability-induced large deformations occurring cyclically during flight. We have found that aerodynamic forces are not essential to trigger the buckling sequence that leads to sound production. Indeed, we have demonstrated that the aeroelastic tymbal can be buckled by direct physical manipulation (quasi-static folding) of an otherwise stationary hindwing. Hence, inertial and elastic forces are more important in the actuation sequence, as these are known to play an important role in driving wing camber changes and bending (34).

While aerodynamic forces are likely less important than inertial forces to trigger tymbal actuation, the fundamental vibrational frequencies of the sound-radiating feature of the wing are influenced by pressure-induced strains. This is one source of uncertainty that can explain differences between measured and modeled sound pressure levels and frequencies. Pressure changes due to vortices around the wing may also influence the sound radiation in the vicinity of the wing, but we believe that this effect is negligible in the far field, where acoustic measurements were made and, indeed, the distance over which the clicking sounds intend to jam a bat’s echolocation systems. As the frequencies of the clicking sound are far greater than the wingbeat frequency, any pressure variations caused by the wingbeat cycle are also considered of negligible importance to the acoustic signal.

This research shows that claval rotation (folding and unfolding of the anal area of the hindwing with respect to the remigium) induces a sequential, cellular buckling mechanism at the interface between the striated band (the corrugated feature of the wing) and the window (the clear, scaleless portion of the wing), thereby inducing ultrasonic clicks by snap-through instabilities of individual striations. We show that the scaleless window is the main sound-radiating structure that undergoes local mechanical vibrations and amplifies the sound produced by the snapping stria at the edge of the window. The obtained resonant frequencies of the window closely match the reported acoustic peak frequencies of tethered moths (11).

The curvature in the tymbals of cicadas is believed to aid in effective sound production (35). Our micro-CT measurements of aeroelastic tymbals have equally highlighted the importance of curvature by revealing cambered wing sections, whose snap-through response we modeled by means of one-dimensional arches. The reduced thickness at the interface between the striated band and the window membrane serves as a hinge, enabling an articulate movement between both segments. Crucially, we have shown that it is possible to produce a sequence of snaps under a single loading condition (claval rotation) by means of sequential stria-by-stria buckling. This indicates that the graded cuticular properties and thickness variations throughout the tymbal are key to enable the buckling behavior. While our simplified analysis demonstrates a feasible phenomenological model of the buckling behavior of individual stria, the spanwise curvature and the presence of local stiffening structures on the wing (intermediate ridges along the tymbal) can also introduce new constraints on the system’s deformation that are not accounted for herein.

The acoustic field was investigated using two vibrating plates based on the shape and projected area of the aeroelastic tymbal, mimicking the full upstroke position of the wings, with vibrations enforced in- and out-of-phase of each other at the peak acoustic frequency. This analysis revealed that even when the acoustic field of a single tymbal induces negligible sound production toward the front and back sides of the moth (due to the dipole condition), it is possible to create frontal acoustic waves due to the interaction and of out-of-phase excitations and reflections created by wing boundaries. Our idealized case shows that reported experimental SL values fall within the range of our acoustic models. The small observed differences could be explained by a larger baffle boundary (size of the wing) due to the presence of the forewing as well as the influence of clap-and-fling mechanisms (26). Therefore, it is possible that during the fling stage (when the wings peel apart), a cavity is created between the hindwings and the clicks are mostly reflected toward the front of the moth. Of course, other sources of acoustic losses are also possible to explain these differences. It is important for future research to experimentally assess, and to model accordingly, the potential sound transmission (through the wing membrane), as well as the impedance and absorption of the wing scales or body bristles (3, 4, 36).

In conclusion, this paper presents a comprehensive multi-physics analysis coupling biological characterization with mechano-acoustic engineering modeling to uncover the complex mechanics governing the buckling-driven sound production of aeroelastic tymbals. While tymbal organs demonstrate the possibility of using buckling for functionality—and ultimately survival—elastic instabilities of this kind are usually avoided in traditional engineering design. More recently, reversible elastic instabilities have been embraced in the field of morphing and adaptive structures (37–40). In particular, buckling is exploited to shift between stable shapes, thereby enabling large deformations in otherwise stiff structures. In fact, several morphing devices (41) have taken inspiration from the bistable response that the Venus fly trap (*Dionaea muscipula*) employs to catch its prey (42). We anticipate that an improved understanding of *Yponomeuta* tymbals will equally inspire biomimetic design of novel acousto-mechanical devices, e.g., for structural health monitoring. In addition, we speculate that an understanding of multiple and sequential instabilities and their coupling to sound production may lead to novel MEMS and/or resonators and energy harvesters.

## Materials and Methods

### CTscan.

A microscale 3D model of the right hindwing of *Y. evonymella* was generated by synchrotron X-ray imaging at the Diamond Light Source facility in the Diamond Manchester Imaging Branchline (I13-2). It was obtained using pink light, a magnification of 2x and exposure time of 20 ms, resulting in a voxel size of 1.6μm. The geometry is analyzed using the software Avizo 9.7 to generate 3D mesh representations of the structure and exported to a stereolithography (*.stl) file. Further post-processing of the *.stl file and dimensioning were carried out using the software NX (Siemens Industry Software Inc.).

### Confocal Microscopy.

Fresh hindwings of *Y. malinellus* and *Y. cagnagella* were mounted individually between two coverslips and embedded in glycerol. The presence and the distribution of four different autofluorescences within the aeroelastic tymbal were analyzed using the confocal laser scanning microscope Leica AOBS SP8 (Leica Microsystems GmbH) attached to a Leica DMi8 inverted epifluorescence microscope equipped with a dry immersion objective lens with magnification 20x (HC PL APO 20x/0.75 CS2) and four solid-state lasers (wavelengths: 405, 488, 555, 639 nm).

### Laser-Doppler Vibrometry.

The dynamic response was measured using a scanning vibrometer PSV-400 (laser Doppler vibrometer, Polytec GmbH) with a mounted microscan lens PSV-A-CL-80. A fresh hindwing cut at the thorax was glued on the face of a piezoceramic column Z6T6D-LYX (C-6), Fuji Ceramics Corporation to dynamically excite the hindwing. A swept sinusoidal chirp signal was applied through a frequency range of 10 to 200 kHz. The frequency response of the aeroelastic tymbal was obtained and analyzed using the VibSoft software, the frequency spectrum was obtained by averaging only the measured points on the area of the aeroelastic tymbal, and the scanning point grid has a scanning step of 0.02 mm. The signal was analyzed using an FFT function with a rectangular window and the modal shapes are post-processed using the ScanViewer package.

### Finite Element Models.

The stability of the curved beam models is evaluated by employing the modified Riks method and also implicit time integration in the Abaqus commercial software package (43). No damping is assigned in the dynamic analysis, as only the verification of the stability of the structure was of interest. However, moderate dissipation was used to improve convergence and reduce output noise (43). A mesh sensitivity analysis was carried out to determine the mesh size for converged results, using a total of 500 B22 beam quadratic elements. The boundary conditions were assumed to be fixed on the side of the cubital vein and pinned with the application of an angular displacement (claval rotation) at the support located at the claval flexion line. The cuticle material was considered isotropic with a Young’s modulus of $E=3\times 10^{9}$ Pa, Poisson’s ratio ν=0.4 and density $\rho_{\text{cuticle}}=1,200\;$kg/m^3^. The studied parameters are shown in Table 1. The total base is the distance between the extreme points of each curved beam of indicated radius. The chord distance is measured from the extreme at the position of the cubital vein to the midpoint of the interface (compliant hinge in the beam). The thicknesses correspond to the striated band ($t_{\text{SB}}$), compliant region ($t_{\text{hinge}}$) and the window segments ($t_{\text{win}}$).

A vibrating plate model obtained from the dorsal projection contour of the aeroelastic tymbal (area $A_{\text{tymbal}}=0.255$ mm^2^, obtained from Fig. 2*A*; see *SI Appendix*, Fig. S5) was used to study the structural-acoustic coupled behavior of the aeroelastic tymbal. The thickness of the tymbal plate is considered uniform (t=6μm) with isotropic material properties and a simply supported boundary condition at the edges. First, the plate is analyzed using a modal dynamic step in Abaqus (43) and the Young’s modulus is adjusted (E=1.81 GPa, ν=0.4, ρ=1,200kg/m^3^) to obtain and match the resonant frequencies obtained through LDV (Fig. 3). Then, considering a damping factor of ζ=0.05 (Rayleigh damping factors, α=4,820, $\beta =2.4\times 10^{-7}$), the plate is subjected to an excitation force capable of inducing the average displacement ($\overset{¯}{U}$) obtained from the curved beam models using the direct integration method in a steady-state dynamic step in Abaqus (43). Each tymbal plate is comprised of a parabolic function (vertex located at x=0, y=0 and z=±0.7 mm) of length 936 μm and width 409 μm covering the measured tymbal area ($A_{\text{tymbal}}$; see *SI Appendix*, Fig. S5*C*). Each plate is embedded in a rectangular acoustic baffle (fluid acceleration ${\ddot{u}}_{f}=0$, *SI Appendix*, Fig. S5 *A* and *B*) of 4.2 mm by 1.4 mm that represents the minimum distance from the tymbal to the closest edges of the wing. The short dimension of the rectangular baffles is aligned at the middle of the tymbal plates and offset a distance x=−0.4 mm with respect to the vertex of the parabola. The distance between rectangular baffles is d=1.4 mm which is equivalent to the distance between both wing joints. Additionally, a baffle boundary representing the top region of the body is enforced between the wing baffles (at x=−0.4 mm, *SI Appendix*, Fig. S5*A*). The plate was modeled using quadrilateral S4R shell elements (1,800 elements in total). A spherical volume composed of two semi-spheres of radius $R_{\text{sphere}}=0.02\;$m (radius of approximately one and a half-wavelengths for the lower frequency) was employed to model the acoustic domain. The semi-sphere volume was meshed using solid acoustic elements (AC3D4, ≈3,000,000 elements in total), with air at standard conditions ($c_{\text{air}}=343\;$m/s, $\rho_{\text{air}}=1.21\;$kg/m^3^, and K=142,355Pa). The tymbal sound radiation is an exterior acoustic problem; therefore, the surfaces of the semi-spheres were assigned ACIN3D3 infinite acoustic elements to mitigate acoustic wave reflections at the sphere’s surface boundary. The structural-acoustic coupling was enforced using tie-constraints between the shell surfaces and the surface of the top and bottom semi-spherical volumes in contact with the structure. In regions between the acoustic volumes, where acoustic waves were allowed to propagate, an acoustic-acoustic coupling condition was enforced using tie constraints. Also, at regions of the interior volume where a reflective condition (rigid baffle) was required, a zero fluid acceleration boundary condition was enforced. The acoustic pressure from the original sphere was projected to the reference distance r=0.1m using the Abaqus script acousticVisualization.py (44). Finally, the peak-to-peak SL was obtained using

$$\begin{array}{c} \;\text{SL}=20\log_{10}\left[\frac{|p_{1}-p_{2}|}{2\sqrt{2}P_{\text{ref}}}\right], \end{array}$$

where $p_{1}-p_{2}$ is a pressure range and the reference pressure $P_{\text{ref}}=20\;\mu$Pa.

## Supplementary Material

Appendix 01 (PDF)

Movie S1. Lateral view of a tethered *Yponomeuta* producing clicks without clapping the wings (1).

Movie S2. Dorsal view of the sequential buckling on the left aeroelastic tymbal of *Yponomeuta* (indicated during two buckling cycles), when mounted as shown in Fig. S1 and subjected to claval rotation (the direction of buckling propagation is indicated). The sound is recorded from a ultra sound advice mini-3 bat detector at 25 kHz (1). SB – striated band, CR – claval rotation, CFL – claval flexion line, Cu – cubital.

## Data Availability

Acoustic model; Beam models; Confocal data; LDV data - resonance spectra; .stl geometries data have been deposited in data.bris.ac.uk (27).

## References

[1] H. M. ter Hofstede, J. M. Ratcliffe, Evolutionary escalation: The bat–moth arms race. J. Exp. Biol. **219**, 1589–1602 (2016). 27252453 10.1242/jeb.086686

[2] T. R. Neil, E. E. Kennedy, B. J. Harris, M. W. Holderied, Wingtip folds and ripples on saturniid moths create decoy echoes against bat biosonar. Curr. Biol. **31**, 4824–4830.e3 (2021). 34506731 10.1016/j.cub.2021.08.038

[3] Z. Shen, T. R. Neil, D. Robert, B. W. Drinkwater, M. W. Holderied, Biomechanics of a moth scale at ultrasonic frequencies. Proc. Natl. Acad. Sci. U.S.A. **115**, 12200–12205 (2018). 30420499 10.1073/pnas.1810025115 PMC6275474

[4] T. R. Neil, Z. Shen, D. Robert, B. W. Drinkwater, M. W. Holderied, Thoracic scales of moths as a stealth coating against bat biosonar. J. R. Soc. Interface **17**, 20190692 (2020). 32093539 10.1098/rsif.2019.0692 PMC7061704

[5] T. R. Neil, Z. Shen, D. Robert, B. W. Drinkwater, M. W. Holderied, Moth wings are acoustic metamaterials. Proc. Natl. Acad. Sci. U.S.A. **117**, 31134–31141 (2020). 33229524 10.1073/pnas.2014531117 PMC7733855

[6] J. R. Barber, W. E. Conner, Acoustic mimicry in a predator–prey interaction. Proc. Natl. Acad. Sci. U.S.A. **104**, 9331–9334 (2007). 17517637 10.1073/pnas.0703627104 PMC1890494

[7] N. J. Dowdy, W. E. Conner, Acoustic aposematism and evasive action in select chemically defended arctiine (Lepidoptera: Erebidae) species: Nonchalant or not? PLoS One **11**, 1–20 (2016). 10.1371/journal.pone.0152981 PMC4838332 27096408

[8] A. J. Corcoran, J. R. Barber, W. E. Conner, Tiger moth jams bat sonar. Science **325**, 325–327 (2009). 19608920 10.1126/science.1174096

[9] A. Y. Kawahara, J. R. Barber, Tempo and mode of antibat ultrasound production and sonar jamming in the diverse hawkmoth radiation. Proc. Natl. Acad. Sci. U.S.A. **112**, 6407–6412 (2015). 25941377 10.1073/pnas.1416679112 PMC4443353

[10] A. D. Blest, T. S. Collett, J. D. Pye, P. B. Medawar, The generation of ultrasonic signals by a new world arctiid moth. Proc. R. Soc. London. Ser. B. Biol. Sci. **158**, 196–207 (1963).

[11] L. J. O’Reilly, D. J. L. Agassiz, T. R. Neil, M. W. Holderied, Deaf moths employ acoustic Müllerian mimicry against bats using wingbeat-powered tymbals. Sci. Rep. **9**, 1444 (2019). 30723216 10.1038/s41598-018-37812-z PMC6363749

[12] J. R. Barber, W. E. Conner, Tiger moth responses to a simulated bat attack: Timing and duty cycle. J. Exp. Biol. **209**, 2637–2650 (2006). 16809455 10.1242/jeb.02295

[13] W. Conner, “Un chant d’appel amoureux’’: Acoustic communication in moths. J. Exp. Biol. **202**, 1711–1723 (1999). 10359675 10.1242/jeb.202.13.1711

[14] L. J. O’Reilly, B. J. Harris, D. J. L. Agassiz, M. W. Holderied, Convergent evolution of wingbeat-powered anti-bat ultrasound in the microlepidoptera. Front. Ecol. Evol. **9**, 648223 (2021).

[15] R. Wootton, The geometry and mechanics of insect wing deformations in flight: A modelling approach. Insects **11**, 446 (2020). 32709085 10.3390/insects11070446 PMC7412480

[16] J. H. Fullard, B. Heller, Functional organization of the arctiid moth tymbal (Insecta, Lepidoptera). J. Morphol. **204**, 57–65 (1990). 29865714 10.1002/jmor.1052040107

[17] W. J. Wolfgang, L. M. Riddiford, Cuticular morphogenesis during continuous growth of the final instar larva of a moth. Tissue Cell **13**, 757–772 (1981). 7330856 10.1016/s0040-8166(81)80012-2

[18] E. Appel, L. Heepe, C. P. Lin, S. N. Gorb, Ultrastructure of dragonfly wing veins: Composite structure of fibrous material supplemented by resilin. J. Anatomy **227**, 561–582 (2015). 10.1111/joa.12362 PMC4580113 26352411

[19] R. J. Wootton, Support and deformability in insect wings. J. Zool. **193**, 447–468 (1981).

[20] Y. Ma, J. G. Ning, H. L. Ren, P. F. Zhang, H. Y. Zhao, The function of resilin in honeybee wings. J. Exp. Biol. **218**, 2136–2142 (2015). 25987733 10.1242/jeb.117325

[21] J. A. Faber, A. F. Arrieta, A. R. Studart, Bioinspired spring origami. Science **359**, 1386–1391 (2018). 29567709 10.1126/science.aap7753

[22] N. Skals, A. Surlykke, Sound production by abdominal tymbal organs in two moth species: The green silver-line and the scarce silver-line (noctuoidea: Nolidae: Chloephorinae). J. Exp. Biol. **202**, 2937–2949 (1999). 10518475 10.1242/jeb.202.21.2937

[23] M. Jensen, T. Weis-Fogh, J. W. S. Pringle, Biology and physics of locust flight. V. Strength and elasticity of locust cuticle. Philos. Trans. R. Soc. London. Ser. B Biol. Sci. **245**, 137–169 (1962).

[24] J. Michels, S. Gorb, Detailed three-dimensional visualization of resilin in the exoskeleton of arthropods using confocal laser scanning microscopy. J. Microscopy **245**, 1–16 (2012). 10.1111/j.1365-2818.2011.03523.x 22142031

[25] H. Peisker, J. Michels, S. N. Gorb, Evidence for a material gradient in the adhesive tarsal setae of the ladybird beetle *Coccinella septempunctata*. Nat. Commun. **4**, 1661 (2013). 23552076 10.1038/ncomms2576

[26] T. Weis-Fogh, Quick estimates of flight fitness in hovering animals, including novel mechanisms for lift production. J. Exp. Biol. **59**, 169–230 (1973).

[27] A. Pirrera, R. Groh, H. Mendoza Nava, M. Holderied, Data for buckling-induced sound production in the aeroelastic tymbals of *Yponomeuta* (2023). data.bris Research Data Repository. 10.5523/bris.m9otxdltertz1zmk8jgmwmcxg. PMC10873622 38315846

[28] G. W. Hunt, R. M. J. Groh, T. J. Dodwell, Maxwell tipping points: The hidden mechanics of an axially compressed cylindrical shell. Proc. R. Soc. A: Math. Phys. Eng. Sci. **476**, 20200273 (2020). 10.1098/rspa.2020.0273 PMC7544337 33061790

[29] D. J. L. Agassiz, Do small ermine moths sing? Possible stridulatory sound production in Yponomeutidae (lepidoptera) J. Nat. Hist. **51**, 1229–1236 (2017).

[30] F. Montealegre-Z, J. F. C. Windmill, G. K. Morris, D. Robert, Mechanical phase shifters for coherent acoustic radiation in the stridulating wings of crickets: The plectrum mechanism. J. Exp. Biol. **212**, 257–269 (2009). 19112145 10.1242/jeb.022731

[31] L. E. Kinsler, A. R. Frey, A. B. Coppens, J. V. Sanders, Fundamentals of Acoustics (Wiley, ed. 4, 2000).

[32] T. Nimura, Y. Watanabe, Effect of a finite circular baffle board on acoustic radiation. J. Acoust. Soc. Am. **25**, 76–80 (1953).

[33] D. A. Russell, On the sound field radiated by a tuning fork. Am. J. Phys. **68**, 1139–1145 (2000).

[34] S. A. Combes, T. L. Daniel, Into thin air: Contributions of aerodynamic and inertial-elastic forces to wing bending in the hawkmoth *Manduca sexta*. J. Exp. Biol. **206**, 2999–3006 (2003). 12878668 10.1242/jeb.00502

[35] J. W. S. Pringle, A physiological analysis of cicada song. J. Exp. Biol. **31**, 525–560 (1954).

[36] J. Zeng , Moth wing scales slightly increase the absorbance of bat echolocation calls. PLoS One **6**, 1–6 (2011). 10.1371/journal.pone.0027190 PMC3212535 22096534

[37] G. Arena , Adaptive compliant structures for flow regulation. Proc. R. Soc. A: Math. Phys. Eng. Sci. **473**, 20170334 (2017). 10.1098/rspa.2017.0334 PMC5582188 28878567

[38] D. P. Holmes, Elasticity and stability of shape-shifting structures. Curr. Opin. Colloid Interface Sci. **40**, 118–137, Particle Systems (2019).

[39] A. R. Champneys , Happy catastrophe: Recent progress in analysis and exploitation of elastic instability. Front. Appl. Math. Stat. **5**, 34 (2019).

[40] J. Shen , Active reconfiguration of multistable metamaterials for linear locomotion. Phys. Rev. B **107**, 214103 (2023).

[41] F. J. Esser, P. Auth, T. Speck, Artificial venus flytraps: A research review and outlook on their importance for novel bioinspired materials systems. Front. Robot. AI **7**, 75 (2020). 33501242 10.3389/frobt.2020.00075 PMC7806029

[42] Y. Forterre, J. M. Skotheim, J. Dumais, L. Mahadevan, How the Venus flytrap snaps. Nature **433**, 421–425 (2005). 15674293 10.1038/nature03185

[43] D. Systèmes, Abaqus 6.12, Abaqus/CAE User’s Manual (Dassault Systèmes Simulia Corp., Providence, RI, USA, 2012).

[44] D. Systèmes, Abaqus 6.11, Scripting User’s Manual (Dassault Systèmes Simulia Corp, Providence, RI, USA, 2011).

