# Supplementary material for: Buckling-induced sound production in the aeroelastic tymbals of Yponomeuta
Source: Proc Natl Acad Sci U S A. 2024 Feb 5;121(7):e2313549121. doi: 10.1073/pnas.2313549121 (PMC10873622; doi:10.1073/pnas.2313549121)
Supplement: Supplementary file 1 — Appendix 01 (PDF) [file pnas.2313549121.sapp.pdf]

Supplementary Information for

Buckling-induced sound production in the aeroelastic tymbals of *Yponomeuta*

Hernaldo Mendoza Nava, Marc W. Holderied, Alberto Pirrera, Rainer M. J. Groh

Marc W. Holderied.
E-mail: marc.holderied@bristol.ac.uk

This PDF file includes:

Figs. S1 to S6 (not allowed for Brief Reports)
Table S1 (not allowed for Brief Reports)
Legends for Movies S1 to S2
SI References

Other supplementary materials for this manuscript include the following:

Movies S1 to S2

1. Mechanically enforced claval rotation to induce train of clicks

To trigger the buckling of striations of the aeroelastic tymbal, claval rotation was applied mechanically in a static, isolated moth wing. By fixing the wing, aerodynamic considerations could be neglected and claval rotation could be isolated as the key mechanism to trigger sequential buckling of the tymbal striations. As shown in Fig. S1, a deceased moth was fixed by an insect pin and one outstretched wing was fixed in place with two pins. The main role of these pins was to lock the movement of the remigium. A moveable pin then pushes on the ventral side of the hindwing to induce negative claval rotation, thereby inducing one train of clicks. By removing the moveable pin, claval rotation is automatically undone, triggering a second train of clicks. A video of this procedure is provided in Movie S2.

2. 3D surface measurement of the buckled and relaxed tymbal

Optical 3D surface measurements of the aeroelastic tymbal were obtained using an Alicona InfiniteFocus G5 microscope (Alicona Imaging GmbH, X-Y step size: $2.6\,\mu$m and Z step size: $5\,\mu$m). The surface plots are imported and post-processed using Siemens NX (Siemens Digital Industries Software). The surface plot of the buckled tymbal (corresponding to the buckled surface in Fig. 2B of the main text) is shown in Fig. S2 and shows multiple parallel profile curves (black) drawn on top of the buckled aeroelastic tymbal. The profile curves of a partially relaxed (unbuckled) tymbal surface are also shown by the blue profile lines to be able to compare them to the buckled tymbal surface. Both buckled and unbuckled surfaces were aligned at the cubital vein (highlighted by Cu).

3. Profile curves of the buckled tymbal

The morphological changes between the buckled and unbuckled tymbal surfaces are further highlighted by directly comparing 12 (of the 13) profile sections highlighted in Fig. S2. The 1D profiles curves are extracted and their Z-axis profiles plotted in Fig. S3. To aid understanding and comparison, the profile curves of the buckled tymbal and the relaxed tymbal are shown on the same set of axes.

4. Table summarising buckled *vs* unbuckled configuration

The maximum transverse displacement of the three curved beam models discussed in the main text (modelled at Stations $X1$–$X3$ shown in Fig. 2A of the main text), with maximum transverse displacement occurring at the compliant hinge, and the spatially averaged displacement during snap-down (from the limit point to the restabilised position) are shown in Table S1. In addition, the maximum displacement at the interface between the striated band and the window obtained from the Alicona curve profiles at the 13 indicated profile sections (see Fig. S2) computed as the maximum transverse distance between the buckled and relaxed profile curves are also listed for comparison. The maximum displacements of the simulated beam models are of the correct order of magnitude, with differences readily explainable by uncertainties regarding material properties, the one-dimensional nature of our beam models and difficulties in freely manipulating the wing under the Alicona microscope.

5. Vibrational excitation of an isolated moth wing

To excite a moth wing and investigate the dominant vibrating portion of the wing, a single wing was attached to a piezoelectric column as shown in Fig. S4. Vibrations of the piezoelectric column thereby excited the entire wing and laser doppler vibrometry measurements identified the dominant vibrating portions of the wing, the vibrational mode shapes and fundamental frequencies. These forced vibration tests and the observations made therein support the assumption of the double plate acoustic models discussed below, that the tymbal window can be assumed to be the dominant sound emitting source, with the rest of the wing considered as a rigid baffle boundary.

6. Double plate acoustic model

To investigate the interaction of two vibrating tymbals, a double plate structural-acoustic model is described in the main text. Two flat plates of a parabolic shape are embedded within a rectangular baffle boundary that represents the embedding of the tymbal window (the main vibrating structure) within the otherwise rigid wings. Details of this model are shown in Fig. S5, with panels A & B showing frontal and lateral views of the tymbal (in yellow) embedded within the rectangular boundary. In both panels the acoustic elements chosen in Abaqus CAE and the applied boundary conditions are depicted. Panel C shows the dimensions of the idealised tymbal area, whose area magnitude is based on measurements made during the biological characterisation described in the main text.

7. Curved beam model

To investigate the morphological change and snap-through response of the curved tymbal structure, 1D cross-sections of the wing (see also Fig. S2) were analysed and idealised as two beam segments with an interstitial compliant hinge. To simulate claval rotation, an angular rotation was then applied to one end of the beam with boundary conditions shown in Fig. S6. To aid understanding of the dimensions shown in Table 1 of the main text, a schematic of the model is shown in Fig. S6.

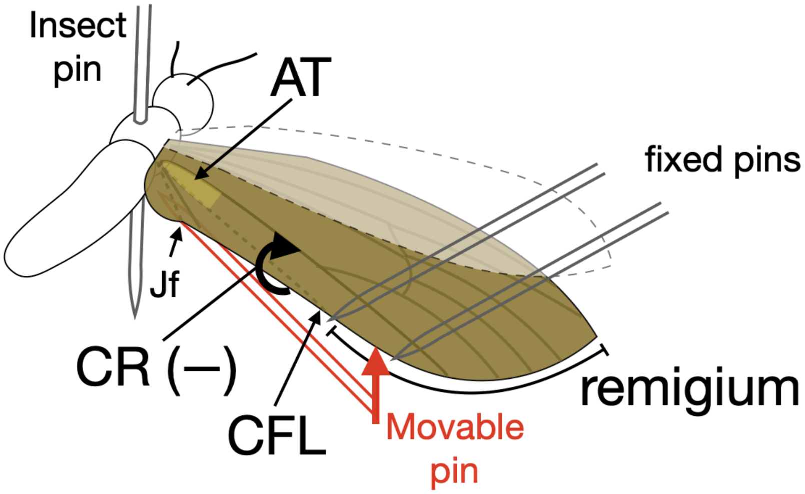

Fig. S1. Diagram of a pinned *Yponomeuta* with two insect pins locking the movement of the remigium on the hindwing when a pin (red) pushes the ventral side of the hindwing. The resulting negative claval rotation (CR) of the anal area from the claval flexion line (CFL) leads to the actuation of the aeroelastic tymbal (AT). The pin movement is applied through a standard manual control manipulator (MM-33, Warner instruments LLC). Jf – jugal fold.

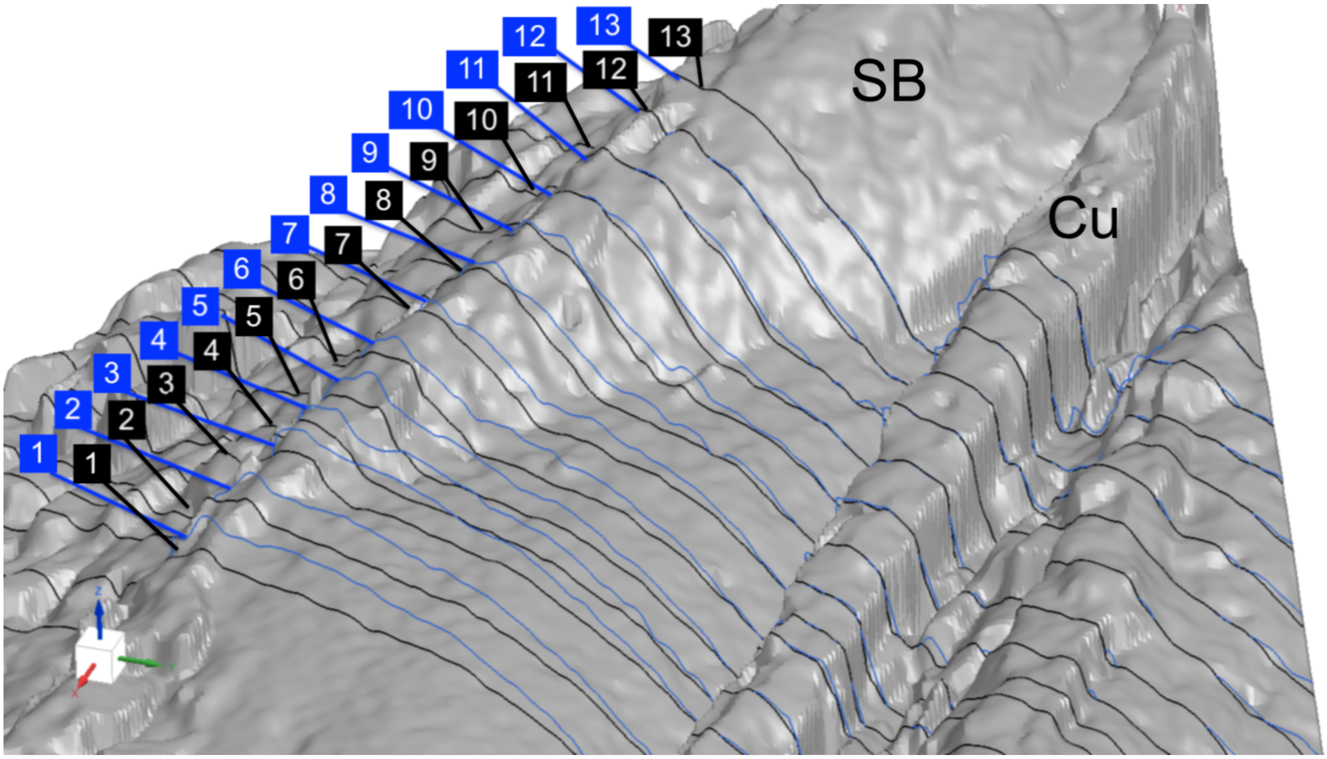

Fig. S2. Surface of the buckled aeroelastic tymbal with individual profile curves of the buckled surface (black) and curves of the partially relaxed (blue) aeroelastic tymbal at multiple stations.

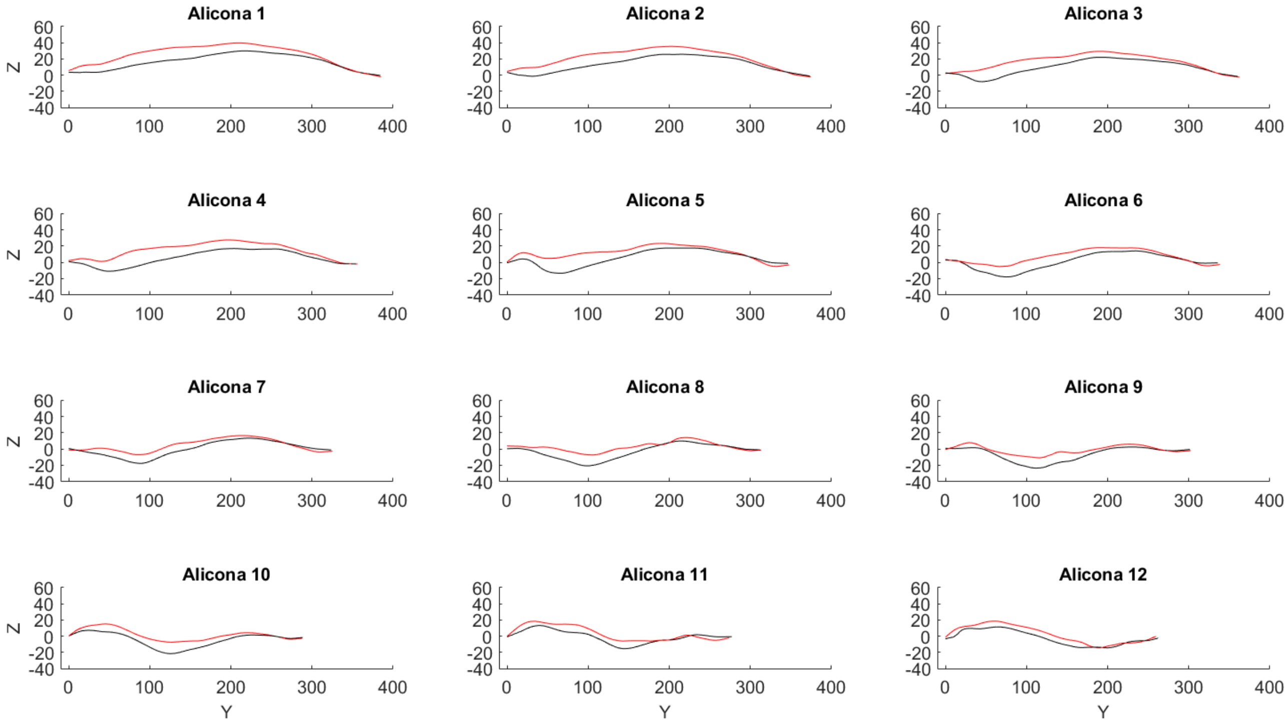

Fig. S3. Profile curves of the buckled (black) and partially relaxed (red) aeroelastic tymbal at the stations indicated in Fig. S2. The start- and end-points of the curves are individually aligned for each curve. All units in μm.

Table S1. Nodal (spatial) average and local maximum displacement of the curved beam models (Stations $X1$–$X3$ noted in Fig. 2A of the main text), and maximum displacement at the interface between the striated band and the window obtained from the Alicona curve profiles indicated in Fig. S2. $\bar{U}_{\mathbf{peak}}$ is the nodally averaged displacement during snap-down (limit point to restabilised position) and $U_{\mathbf{max}}$ is the local maximum displacement between the buckled and unbuckled configurations.

Variable Name	$\bar{U}_{\mathrm{peak}}$	U_{max}	Units
Station $X1$	5.0	37.6	μm
Station $X2$	4.5	25.5	μm
Station $X3$	3.0	27.4	μm
Alicona 1	-	15.1	μm
Alicona 2	-	14.3	μm
Alicona 3	-	17.3	μm
Alicona 4	-	19.9	μm
Alicona 5	-	20.3	μm
Alicona 6	-	16.3	μm
Alicona 7	-	10.9	μm
Alicona 8	-	13.7	μm
Alicona 9	-	13.3	μm
Alicona 10	-	14.2	μm
Alicona 11	-	9.9	μm
Alicona 12	-	8.2	μm
Alicona 13	-	4.5	μm

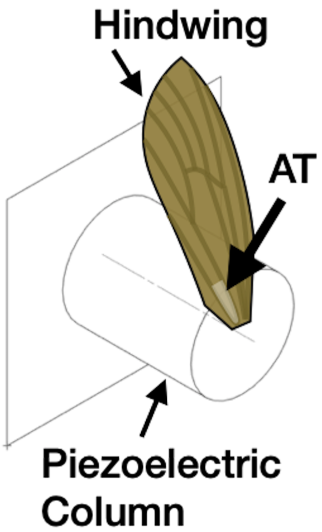

Fig. S4. Diagram of a hindwing mounted on a piezoelectric column. AT – aeroelastic tymbal.

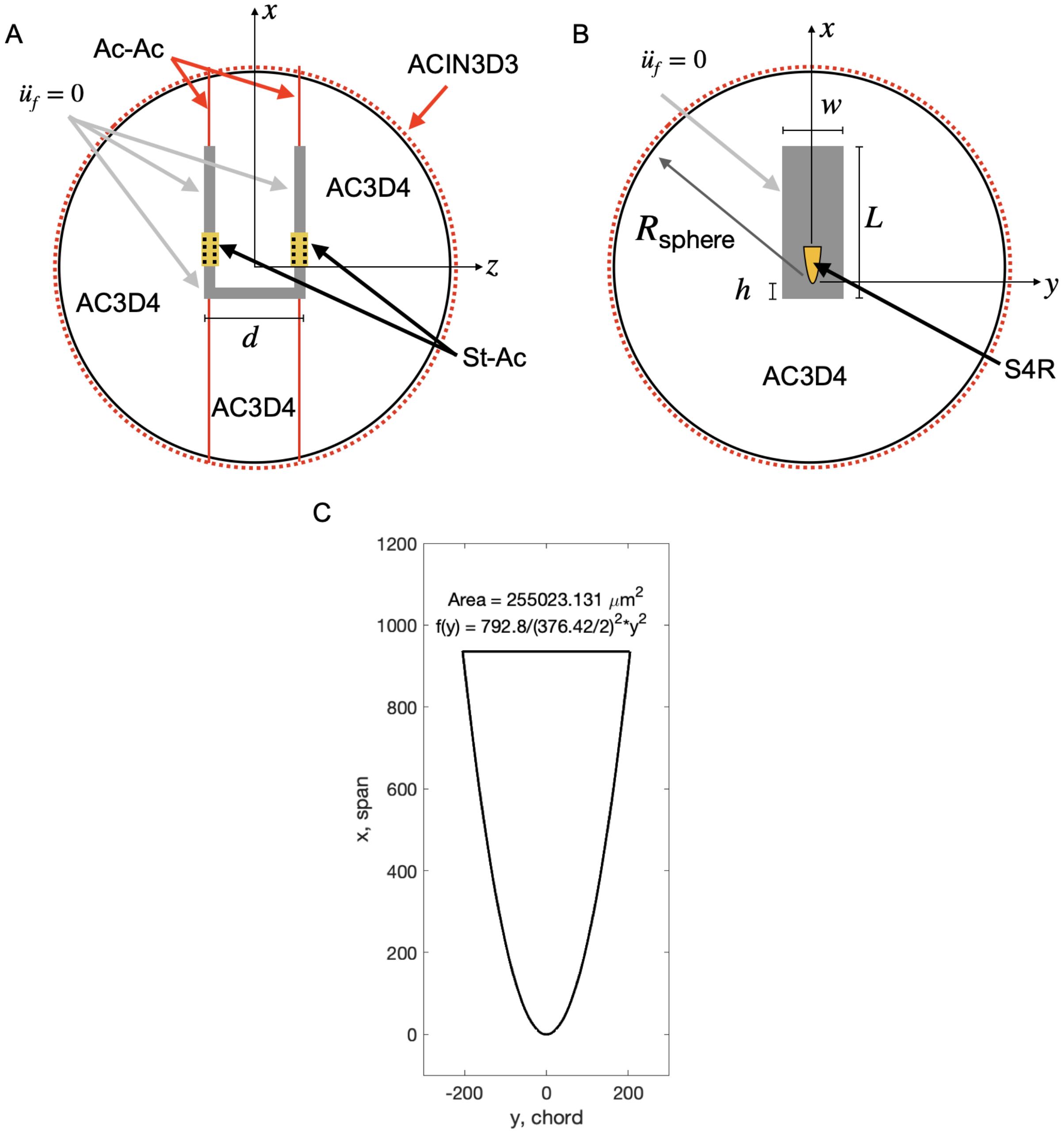

Fig. S5. Schematic of the coupled structural-acoustic model of two parallel tymbal plates embedded in a rectangular baffle boundary that portrays the shortest distance to the closest edges of the wing. (A) Frontal ($d = 1.4$ mm) and (B) lateral planes of the structural-acoustic coupled model of *Yponomeuta* sound radiation ($w = 1.4$ mm, $L = 4.2$ mm, $h = 0.4$ mm, $R_{\text{sphere}} = 0.02$ m). The black circle denotes the exterior of the spherical volume indicating the interior 3D acoustic elements (AC3D4). The surrounding red-dotted line indicates the infinite acoustic elements (ACIN3D3). The black-dotted lines indicate the structural-acoustic coupling (St-Ac) between the vibrating plates (S4R, quadrilateral shell elements) and the volume. The red lines indicate the surfaces between the three solid volumes comprising the sphere subjected to acoustic-acoustic coupling (Ac-Ac). (C) Quadratic function adjusted to the shape and measured area of the aeroelastic tymbal (units in μm.). $\ddot{u}_f$ – fluid acceleration.

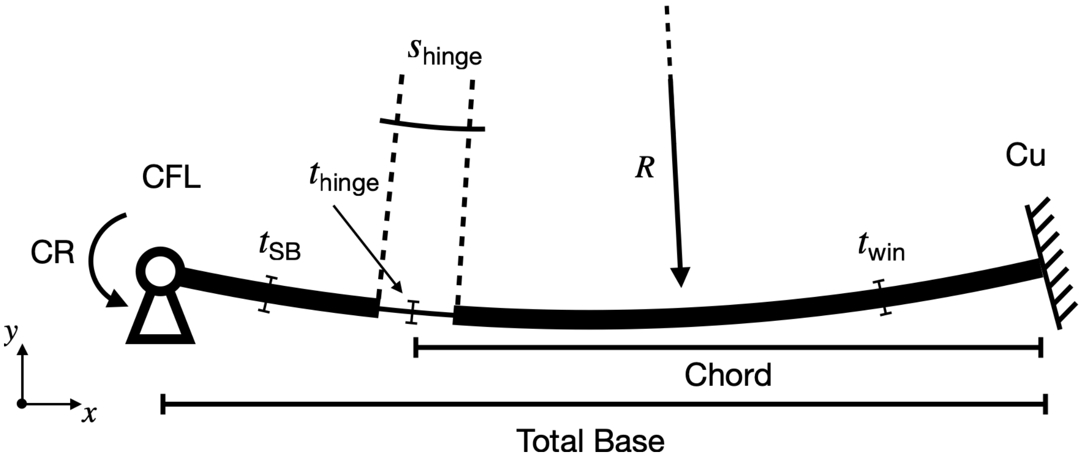

Fig. S6. Diagram of a curved beam of radius R indicating the variable dimensions from Table 1 in the main text. Cu – Cubital vein position, CFL – claval flexion line position, CR – claval rotation. s_{hinge} – length of the hinge section, t_{SB} – thickness of the striated band segment, t_{hinge} – thickness of the hinge section, t_{win} – thickness of the window segment.

Movie S1. Lateral view of a tethered *Yponomeuta* producing clicks without clapping the wings (1).

Movie S2. Dorsal view of the sequential buckling on the left aeroelastic tymbal of *Yponomeuta* (indicated during two buckling cycles), when mounted as shown in Fig. S1 and subjected to claval rotation (the direction of buckling propagation is indicated). The sound is recorded from a ultra sound advice mini-3 bat detector at 25 kHz (1). SB – striated band, CR – claval rotation, CFL – claval flexion line, Cu – cubital.

References

1. A Pirrera, R Groh, H Mendoza Nava, M Holderied, Data for buckling-induced sound production in the aeroelastic tymbals of *Yponomeuta* (2023) data.bris Research Data Repository: https://doi.org/10.5523/bris.m9otxdltertz1zmk8jgmwmcxg.
